# Supplementary material for: A collagen glucosyltransferase drives lung adenocarcinoma progression in mice
Source: Commun Biol. 2021 Apr 19;4:482. doi: 10.1038/s42003-021-01982-w (PMC8055892; doi:10.1038/s42003-021-01982-w)
Supplement: Supplementary file 2 — Supplementary Information [file 42003_2021_1982_MOESM2_ESM.pdf]

# Supplementary information for
# A collagen glucosyltransferase drives lung adenocarcinoma progression in mice

Hou-Fu Guo[1#], Neus Bota-Rabassedas[1], Masahiko Terajima[2], B. Leticia Rodriguez[1], Don L. Gibbons[1], Yulong Chen[1], Priyam Banerjee[1], Chi-Lin Tsai[3], Xiaochao Tan[1], Xin Liu[1], Jiang Yu[1], Michal Tokmina-Roszyk[4], Roma Stawikowska[4], Gregg B. Fields[4], Mitchell D. Miller[5], Xiaoyan Wang[3], Juhoon Lee[6,7], Kevin N. Dalby[6,7], Chad J. Creighton[8,9], George N. Phillips, Jr.[5,10], John A. Tainer[3], Mitsuo Yamauchi[2], Jonathan M. Kurie[1]

[1]Department of Thoracic/Head and Neck Medical Oncology, The University of Texas MD Anderson Cancer Center, Houston, Texas 77030, USA. [2]Division of Oral and Craniofacial Health Sciences, Adams School of Dentistry, University of North Carolina at Chapel Hill, Chapel Hill, North Carolina 27599, USA. [3]Department of Molecular and Cellular Oncology, The University of Texas MD Anderson Cancer Center, Houston, Texas 77030, USA. [4]Institute for Human Health & Disease Intervention (I-HEALTH) and Department of Chemistry & Biochemistry, Florida Atlantic University, Jupiter, Florida 33458, USA. [5]Department of Biosciences, Rice University, Houston, Texas 77251, USA. [6]Division of Medicinal Chemistry, Targeted Therapeutic Drug Discovery and Development Program, College of Pharmacy, The University of Texas at Austin, Austin, Texas 78712, USA. [7]Division of Chemical Biology & Medicinal Chemistry, College of Pharmacy, The University of Texas at Austin, Austin, Texas, 78712, USA [8]Department of Medicine, Dan L. Duncan Cancer Center, Baylor College of Medicine, Houston, Texas 77030, USA. [9]Department of Bioinformatics and Computational Biology, The University of Texas MD Anderson Cancer Center, Houston, Texas 77030, USA. [10]Department of Chemistry, Rice University, Houston, Texas 77251, USA.

Correspondence: J.M.K. (jkurie@mdanderson.org),

# current address: Department of Molecular and Cellular Biochemistry, University of Kentucky, Lexington, Kentucky  40536, USA.

**Supplementary Figure 1 | Uncut western blot images for Figure 1a**

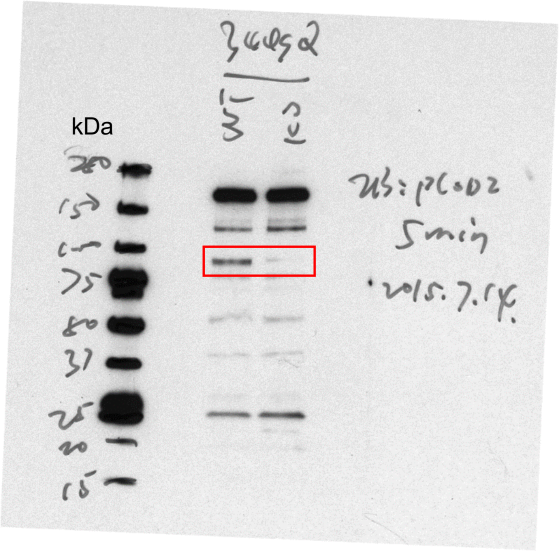


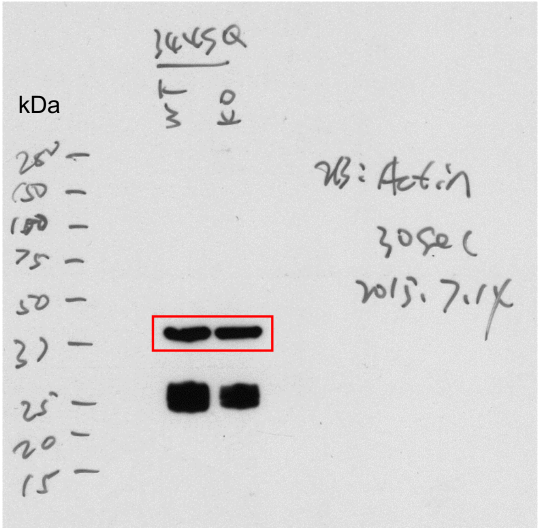

## Supplementary Figure 2 | Uncut western blot images for Figure 2b

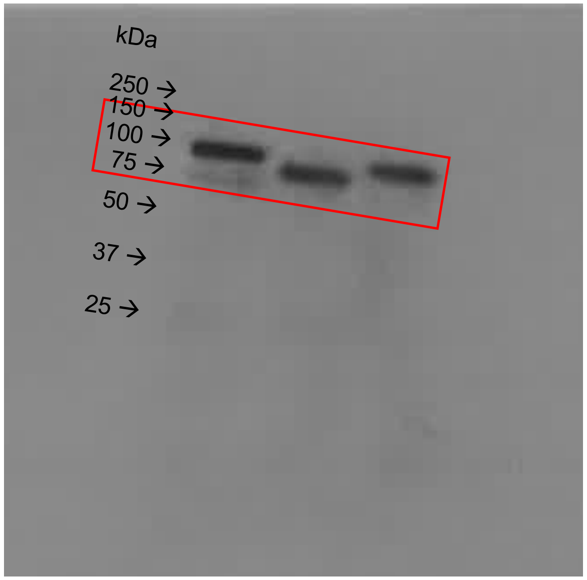


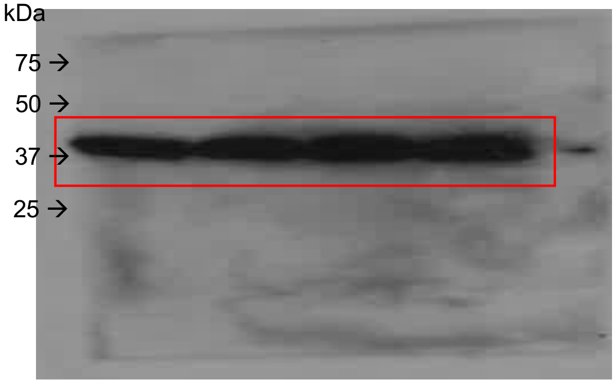

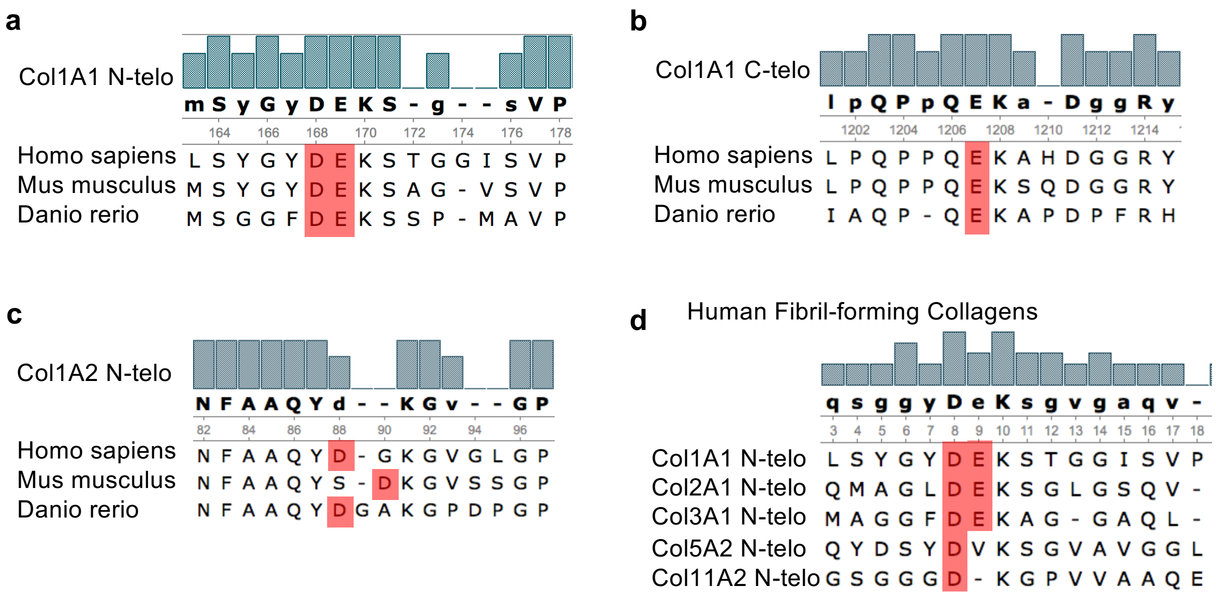


**Supplementary Figure 3 │ Amino acid sequence alignment of collagen's amino (N)- and carboxy (C)-terminal telopeptides identifies acidic residues adjacent to Lys. Amino acid s**equence alignment of vertebrate collagen 1A1 and 1A2 (a-c) and human fibril-forming collagens (1A1, 2A1, 3A1, 5A2, 11A2) (d). Acidic residues at the i-1 and i-2 positions are highlighted in red.

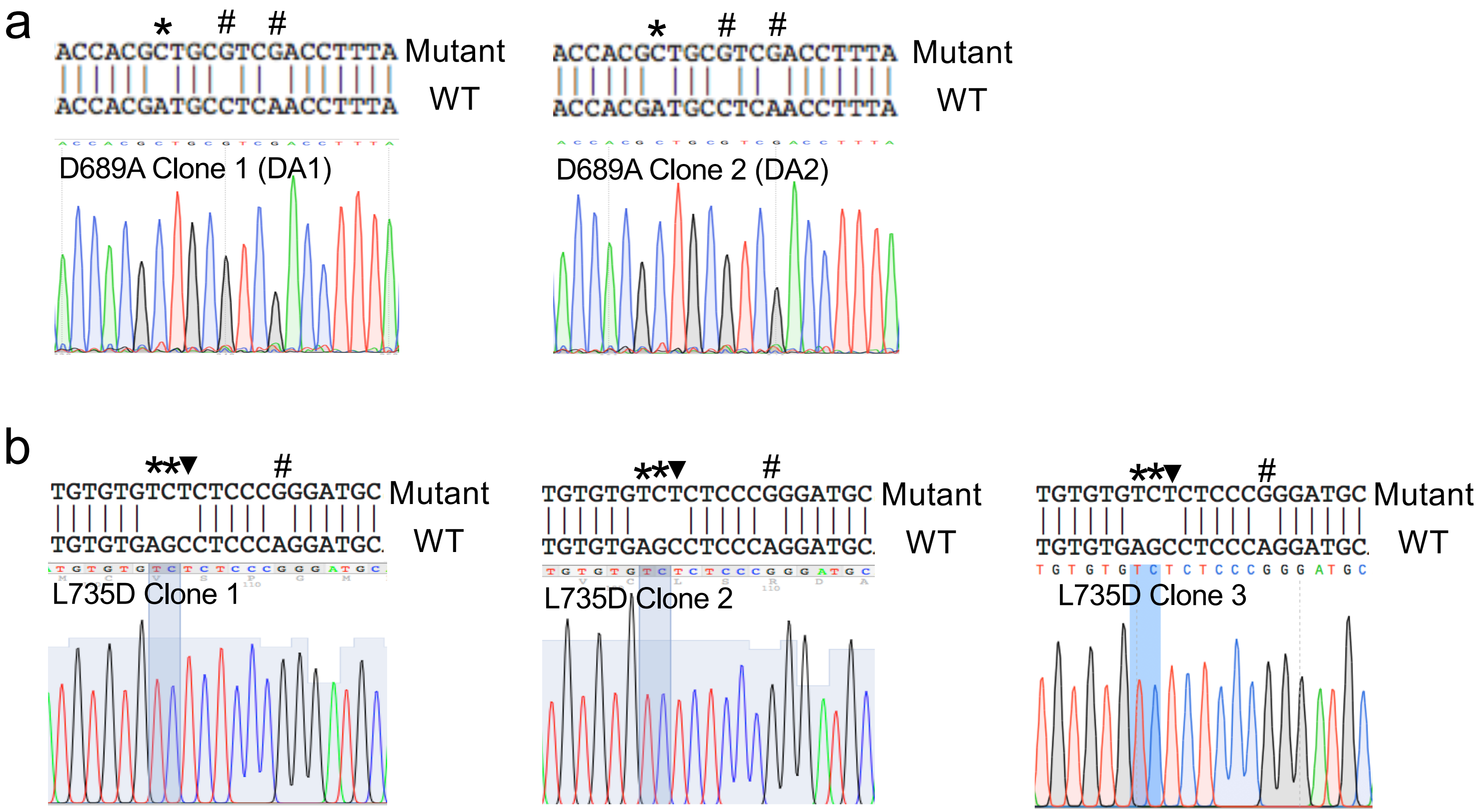


**Supplementary Figure 4 │ Confirmation of CRISPR-Cas9-edited 344SQ cells. a and b,** Once clones grew to acceptable sizes, 344SQ cells were lysed to isolate genomic DNA for PCR amplification and Sanger sequencing. Top: Sequence alignment of *Plod2* alleles in parental and CRISPR/Cas-9-edited 344SQ cells to generate D689A (a) or L735D (b) mutations. Amino acid mutations (*) and silent mutations (#) are indicated. c.2066A>C (D689A). c.2264A>T and c.2265G>C (735D). Silent mutations with Sal1 (a) or SmaI (b) digestion sites were introduced to assist in the identification of positive clones. PAM silent mutation (▼). cDNA sequence chromatograms from homozygous mutant clones are under each panel. Results are from replicate biological samples.

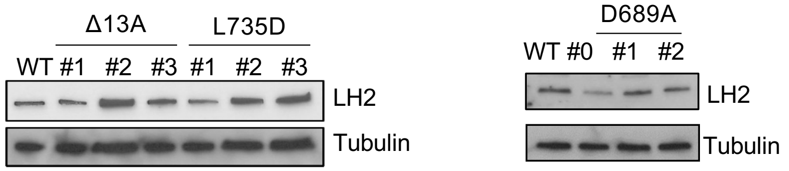


**Supplementary Figure 5 │ *Plod2* mutations do not reduce LH2 protein levels.** LH2/Plod2 protein levels were determined by western blot analysis of parental (WT) and CRISPR/Cas-9-edited 344SQ cells. Tubulin used as loading control. LH2 exon 13A deletion (Δ13A). Dimerization mutant (L735D). $Fe^{2+}$-binding mutant (D689A). LH2D689A clone #0 led to low LH2 protein expression and was removed from further analysis.

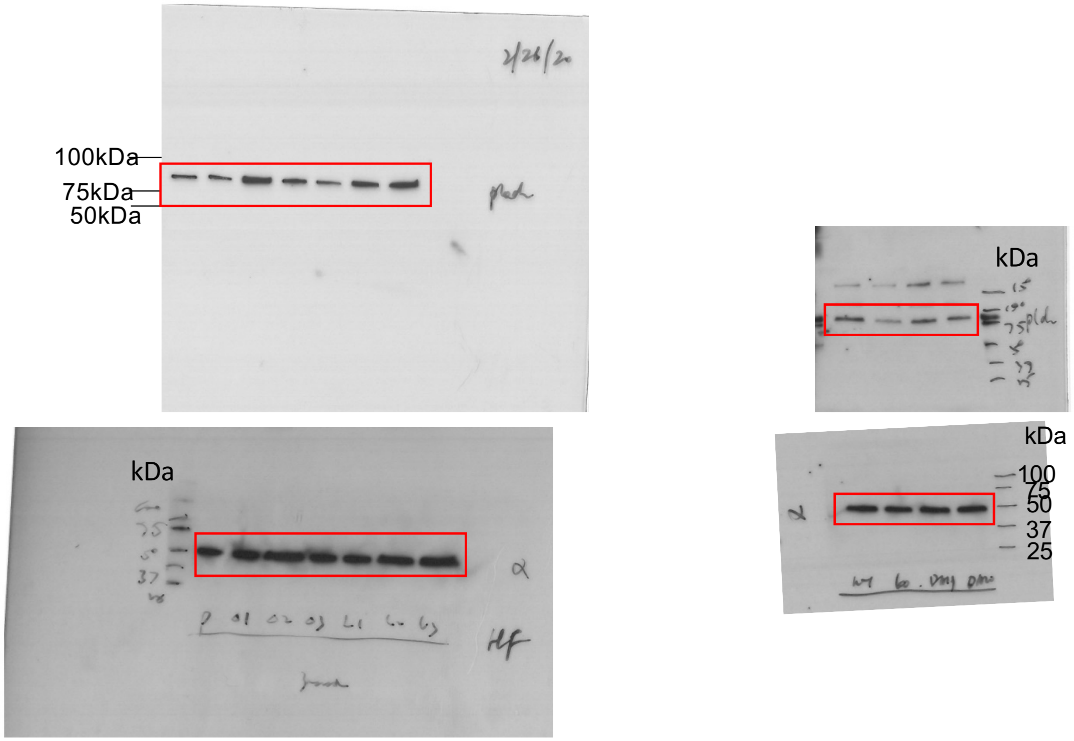

```
mPlod1     --------MRSLLLLAPLAWLLLV------QAKDDAKLEDNLLVLTVATKETEGFRRFKR    46
mPlod2a    MGDRGARPGRLMPMLALLSWAAGLGVA---EETPGRIPADKLLVITVATKENDGFHRFMN    57
mPlod2b    MGDRGARPGRLMPMLALLSWAAGLGVA---EETPGRIPADKLLVITVATKENDGFHRFMN    57
mPlod3     MAAAGPEPRLLLLLLLLLPPLPPVTSASDRPRGANAVNPDKLLVITVATAETEGYRRFLQ    60
                      : :*  *     :               *:***:**** *.:*::** .

mPlod1     SAQFFNYKIQSLGLGEDWSVDGGPAAAGGGQKVRLLKKALEKHADKEDLVILFVDSYDVV    106
mPlod2a    SAKYFNYTVKVLGQGQEWRGGDGMNSIGGGQKVRLLKEAMEHYASQEDLVILFTECFDVV    117
mPlod2b    SAKYFNYTVKVLGQGQEWRGGDGMNSIGGGQKVRLLKEAMEHYASQEDLVILFTECFDVV    117
mPlod3     SAEFFNYTVRTLGLGQEWRGGDVARTVGGGQKVRWLKKEMEKYADQKDMIIMFVDSYDVI    120
           **::***.:: ** *::*       : ******* **: :*::*.::*::*:*.:.:**:

mPlod1     FASGPRELLKKFQQAKSQVVFSAEEHIYPDRRLEAKYPTVPDGKRFLGSGGFIGYAPSLS    166
mPlod2a    FAGGPEEVLKKFQKTNHKIVFAADGLLWPDKRLADKYPVVHIGKRYLNSGGFIGYAPYIS    177
mPlod2b    FAGGPEEVLKKFQKTNHKIVFAADGLLWPDKRLADKYPVVHIGKRYLNSGGFIGYAPYIS    177
mPlod3     LASSPTELLKKFVQSGSHLLFSAESFCWPEWGLAEQYPEVGMGKRFLNSGGFIGFAPTIH    180
           :*..* *:**** ::  :::*:*:   :*:  *  :** *  ***:* ******:** :

mPlod1     KLVAEWEGQDSDSDQLFYTKIFLNPEKREQINISLDHRCRIFQNLDGALDEVVLKFEMGH    226
mPlod2a    RLVQQWNLQDNDDDQLFYTKVYIDPLKREAFNITLDHKCKIFQALNGATDEVVLKFENGK    237
mPlod2b    RLVQQWNLQDNDDDQLFYTKVYIDPLKREAFNITLDHKCKIFQALNGATDEVVLKFENGK    237
mPlod3     QIVRQWNYKDDDDDQLFYTQLYLDPGLREKLKLSLDHKSRIFQNLNGALDEVILKFDQNR    240
           ::* :*: :*.*.******::::*   ** :::***:.:***  *:** ***:***:  :

mPlod1     VRARNLAYDTLPVVVHGNGPTKLQLNYLGNYIPRFWTFETGCTVCDEGLRSLKGIGDEAL    286
mPlod2a    SRVKNTFYETLPVAINGNGPTKILLNYFGNYVPNSWTQENGCALCDVDTIDLSTV--DVP    295
mPlod2b    SRVKNTFYETLPVAINGNGPTKILLNYFGNYVPNSWTQENGCALCDVDTIDLSTV--DVP    295
mPlod3     VRIRNVAYDTLPVVVHGNGPTKLQLNYLGNYVPNGWTPQGGCGFCNQTLRTLPG--GQPP    298
            * :*  *:****.:.******: ***:***:*. ** : ** .*:     *     :

mPlod1     PTVLVGVFIEQPTPFLSLFFLRLLRLRYPQKQMRLFIHNQERHHKLQVEQFLAEHGSEYQ    346
mPlod2a    PKVTLGVFIEQPTPFLPRFLNLLLTLDYPKEALQLFIHNKEVYHEKDIKVFVDKAKHDIS    355
mPlod2b    PKVTLGVFIEQPTPFLPRFLNLLLTLDYPKEALQLFIHNKEVYHEKDIKVFVDKAKHDIS    355
mPlod3     PRVLLAVFVEQPTPFLPRFLQRLLLLDYPPDRISLFLHNSEVYHEPHIADAWPQLQDHFS    358
           * * :.**:*******  *:   ** * **  . : **:**.* :*: .:     :   . .

mPlod1     SVKLVGPEVRMANADARNMGADLCRQDQTCTYYFSVDADVALTEPNSLRLLIEQNKNVIA    406
mPlod2a    SIKIVGPEENLSQAEARNMGMDFCRQDEKCDYYFSVDADVVLTNPRTLKFLIEQNRKIIA    415
mPlod2b    SIKIVGPEENLSQAEARNMGMDFCRQDEKCDYYFSVDADVVLTNPRTLKFLIEQNRKIIA    415
mPlod3     AVKLVGPEEALSAGEARDMAMDSCRQNPECEFYFSLDADAVLTNPETLRVLIEQNRKVIA    418
           ::*:****  :: .:**:*. * ***:  * :***:***..**:*.:*:.*****:::**

mPlod1     PLMTRHGRLWSNFWGGLSADGYYARSEDYVDIVQGRRVGVWNVPYISNIYLIKGSALRAE    466
mPlod2a    PLVTRHGKLWSNFWGALSPDGYYARSEDYVDIVQGNRVGIWNVPYMANVYLIQGKTLRSE    475
mPlod2b    PLVTRHGKLWSNFWGALSPDGYYARSEDYVDIVQGNRVGIWNVPYMANVYLIQGKTLRSE    475
mPlod3     PMLSRHGKLWSNFWGALSPNEYYARSEDYVELVQRKRVGVWNVPYISQAYVIRGETLRTE    478
           *:::***:*******.** : *********::** .***:*****::: *:*:*.:**:*

mPlod1     LQNVDLFHYSKLDSDMSFCANVRQQ---------------------EVFMFLTNRHTFGH    505
mPlod2a    MNERNYFVRDKLDPDMALCRNARDM---------------------GVFMYISNRHEFGR    514
mPlod2b    MNERNYFVRDKLDPDMALCRNARDMTLQREKDSPTPETFQMLSPPKGVFMYISNRHEFGR    535
mPlod3     LPQKEVFSSSDTDPDMAFCKSVRDK---------------------GIFLHLSNQHEFGR    517
           : : : *  .. * **::* ..*:                       :*:.::*:* **:

mPlod1     LLSLDNYQTTHLHNDLWEVFSNPEDWKEKYIHENYTKALAG-KLVETPCPDVYWFPIFTE    564
mPlod2a    LISTANYNTSHLNNDFWQIFENPVDWKEKYINRDYSKIFTE-NIVEQPCPDVFWFPIFSE    573
mPlod2b    LISTANYNTSHLNNDFWQIFENPVDWKEKYINRDYSKIFTE-NIVEQPCPDVFWFPIFSE    594
mPlod3     LLATSRYDTDHLHPDLWQIFDNPVDWREQYIHENYSRALDGEGLVEQPCPDVYWFPLLTE    577
           *::  .*:* **. *:*::*.** **:*:**..:*:: :    :** *****:***:::*

mPlod1     AACDELVEEMEHYGQWSLGDNKDNRIQGGYENVPTIDIHMNQITFEREWHKFLVEYIAPM    624
mPlod2a    RACDELVEEMEHYGKWSGGKHHDSRISGGYENVPTDDIHMKQIGLENVWLHFIREFIAPV    633
mPlod2b    RACDELVEEMEHYGKWSGGKHHDSRISGGYENVPTDDIHMKQIGLENVWLHFIREFIAPV    654
mPlod3     QMCDELVEEMEHYGQWSGGRHEDSRLAGGYENVPTVDIHMKQVGYEDQWLQLLRTYVGPM    637
             ************:** * ..*.*: ******** ****:*:  *  * :::  ::.*:

mPlod1     TEKLYPGYYTRAQFDLAFVVRYKPDEQPSLMPHHDASTFTVNIALNRVGEDYEGGGCRFL    684
mPlod2a    TLKVFAGYYTKGFALLNFVVKYSPERQRSLRPHHDASTFTINIALNNVGEDFQGGGCKFL    693
mPlod2b    TLKVFAGYYTKGFALLNFVVKYSPERQRSLRPHHDASTFTINIALNNVGEDFQGGGCKFL    714
mPlod3     TEYLFPGYHTKTRAVMNFVVRYRPDEQPSLRPHHDSSTFTLNVALNHKGVDYEGGGCRFL    697
           *  :: **:*:    : ***:* *:.* ** ****:****:*:***. * *::****:**

mPlod1     RYNCSVRAPRKGWALLHPGRLTHYHEGLPTTKGTRYIAVSFVDP   728
mPlod2a    RYNCSIESPRKGWSFMHPGRLTHLHEGLPVKNGTRYIAVSFIDP   737
mPlod2b    RYNCSIESPRKGWSFMHPGRLTHLHEGLPVKNGTRYIAVSFIDP   758
mPlod3     RYDCRISSPRKGWALLHPGRLTHYHEGLPTTRGTRYIMVSFVDP   741
           **:* : :*****:::******* *****...***** ***:**
```

**Supplementary Figure 6│ Amino acid sequence alignment of murine *Plod* gene family members.** Mouse LHs/Plods amino acid sequences were aligned. The gene names were labeled on the left and the amino acid numbers on the right. The mPlod2b motif encoded by an alternative spliced exon was colored in red. “*” indicates perfect alignment. “:” and “.” indicate strong and weak similarity, respectively.

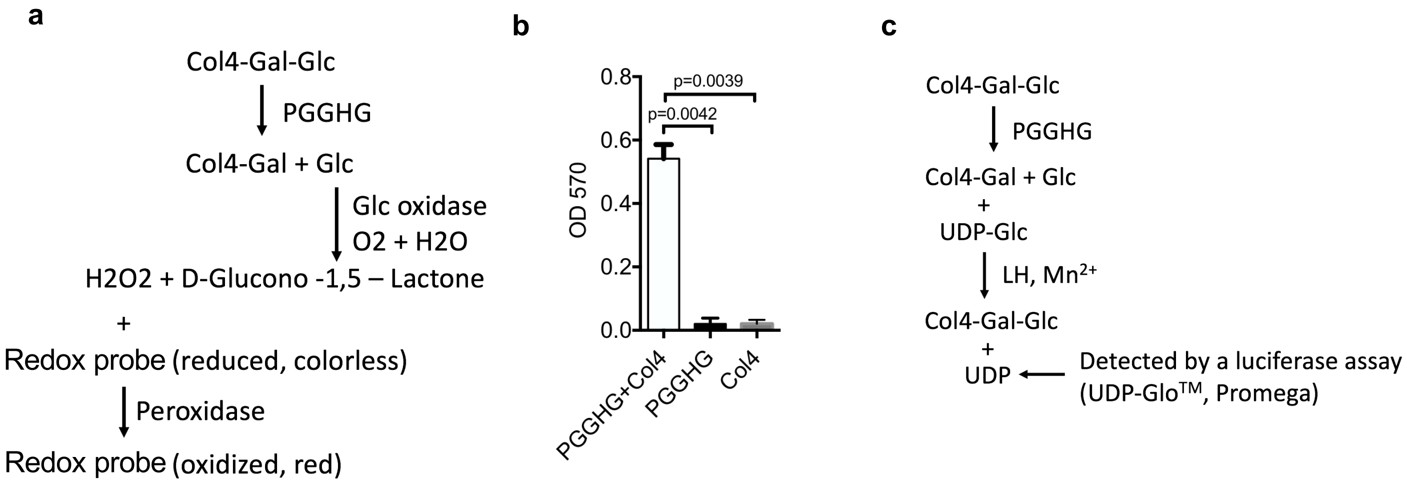


**Supplementary Figure 7│ Preparation of deglucosylated type IV collagen (Col4) by treatment with the putative collagen glucosidase protein-glucosylgalactosylhydroxylysine glucosidase (PGGHG). a,** Schematic illustration of an assay to detect type IV collagen deglucosylation that couples glucose production to redox probe oxidation. Abbreviations not included in text: glucose (Glc), glucosyl-galactosyl-Col4 (Col4-Gal-Glc), galactosyl-Col4 (Col4-Gal). **b,** Implementation of assay in (a) to deglucosylate type IV collagen. Deglucosylation reaction was detected by measuring the absorbance (OD 570) of a redox sensitive dye. Assay reactions detailed in (a). Glc release was not detectable when Col4 or PGGHG was left out of the reaction (n=3). **c,** Schematic illustration of a GGT assay that uses deglucosylated Col4 as substrate. Deglucosylated Col4 (Col4-Gal) produced by PGGHG treatment of glucosylated Col 4 (Col4-Gal-Glc) was reacted with recombinant LH. LH-induced glucosylation was measured by detecting free UDP release with a luciferase assay (UDP-glo$^{TM}$, Promega). Results are expressed as mean ± SDs from triplicate samples. Error bars indicate ±STD. p values, 2-tailed Student's t test.

## Supplementary Figure 8 | Uncut western blot images for Figure 3i

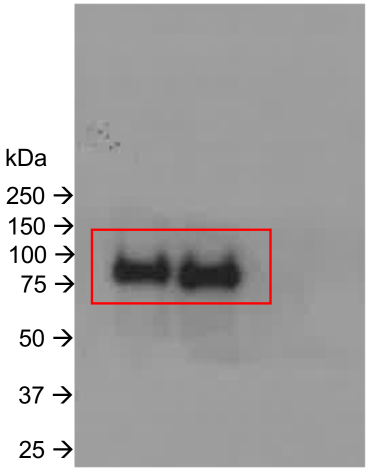


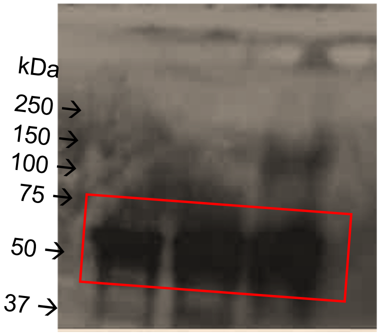

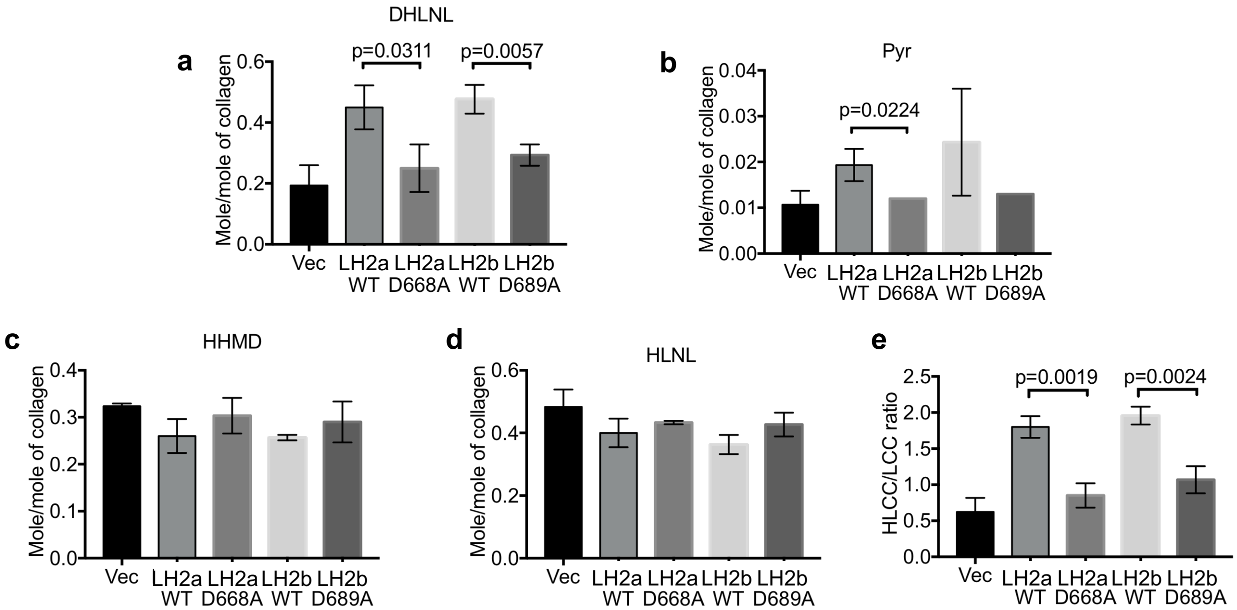


**Supplementary Figure 9 │ Collagen crosslink analysis of MC_shLH2 cells reconstituted with LH2a or LH2b. a-d,** , Collagen cross-link quantification of matrices derived from MC-3T3 (MC) cells that were stably transfected with empty vector (Vec), wild type LH2a (LH2a WT), enzymatically dead LH2a (LH2a D668A), wild type LH2b (LH2b WT) and enzymatically dead LH2b (LH2b D689A). Dihydroxylysinonorleucine (DHLNL, (c), pyridinoline (Pyr, (d), histidinohydroxymerodesmosine (HHMD, (e), hydroxylysinonorleucine (HLNL, (f), and the ratio of HLCCs-to-Lys aldehyde-derived collagen cross-links (LCCs) (g). The HLCC-to-LCC ratio was calculated as (DHLNL+Pyr)/HHMD. Data are the means ± SDs from triplicate samples (n=3). Error bars indicate ±STD. p values, 2-tailed Student t-test.

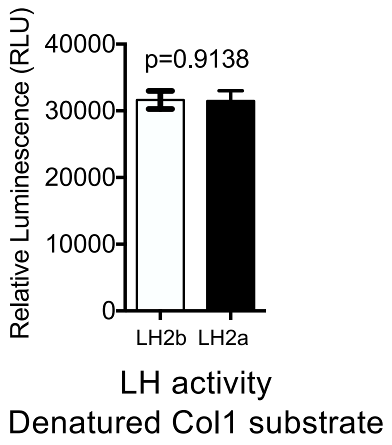


**Supplementary Figure 10 │ Helical LH activity is not regulated by LH2 alternative splicing**. Helical LH activity assay on recombinant LH2a and LH2b using denatured collagen type I as substrate. Lysyl hydroxylase activity was measured by detecting succinate production with an adenosine triphosphate-based luciferase assay. Results are mean values (± S.D.) from triplicate samples (n=3). Error bars indicate ±STD. p values, 2-tailed Student's t test.

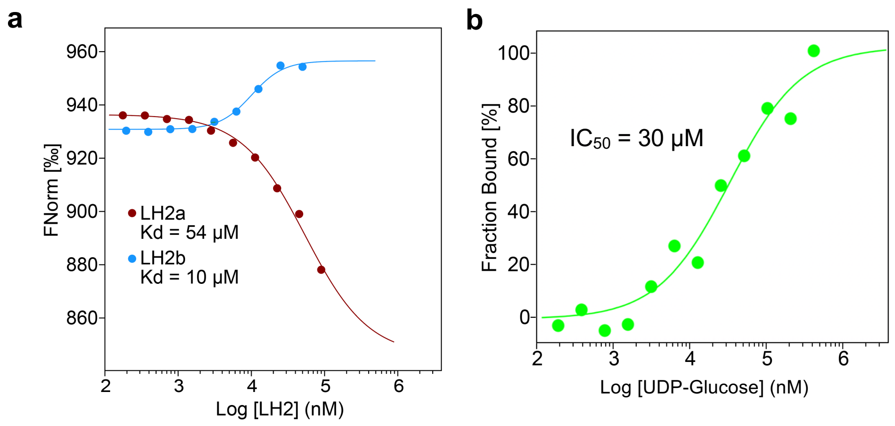


**Supplementary Figure 11 │ UDP-Glc binding determined by microscale thermophoresis.** For binding experiment in a, fixed concentration of fluorescein conjugated UDP-Glucose (50 nM) was titrated with different concentrations of LH2a (red) and LH2b (cyan). For competition assay in b, fixed concentration of fluorescein conjugated UDP-Glucose (50 nM) and LH2b (20 µM) was titrated with different concentrations of unlabeled UDP-Glucose to generate the curves. Curves were used to calculate the $K_d$ values for LH2a (red) and LH2b (cyan) in a and $IC_{50}$ in b. Results are mean values from duplicate samples (n=2).

**Supplementary Figure 12 │ Uncut DNA agarose gel image for Figure 4e**

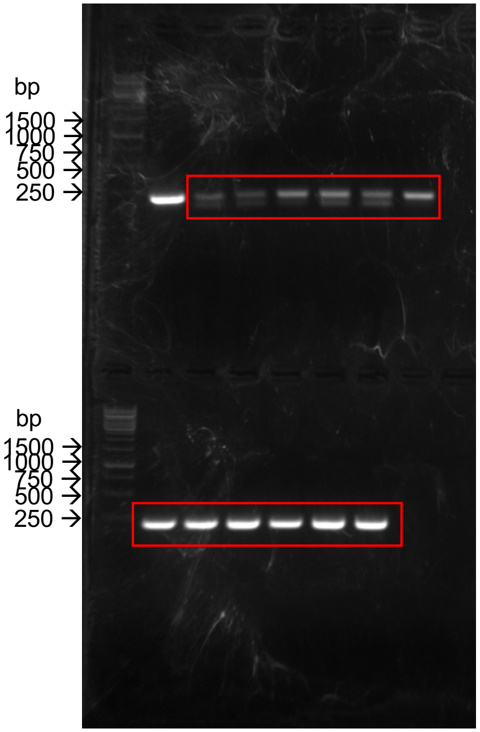

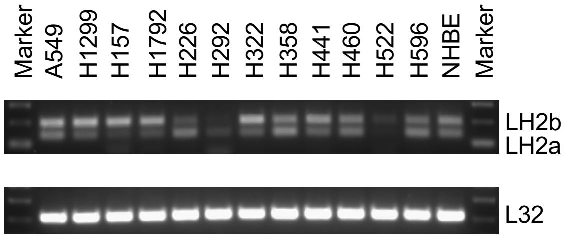


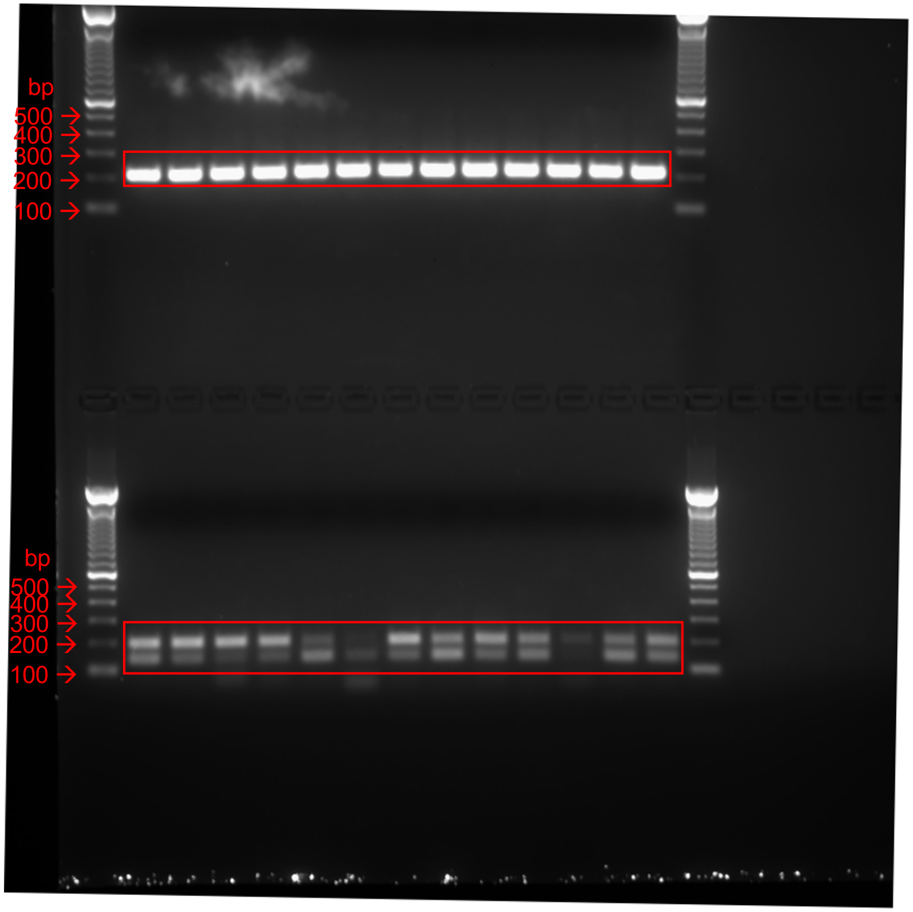


**Supplementary Figure 13 | Reverse Transcriptase-PCR analysis of LH2a and LH2b mRNA levels in human lung adenocarcinoma cell lines**. L32 included as loading control.

| | Poorly metastatic | | Highly metastatic | | | | |
|---|---|---|---|---|---|---|---|
| | 393P | 307P | 344SQ | 344P | 531LN2 | 531LN3 | p value |
| LH2b | 1 | 0.9 | 2.5 | 2.7 | 1.9 | 3 | 0.0108 |
| LH2a | 0.4 | 0.4 | 0.1 | 0.8 | 0.9 | 0.05 | 0.86202 |
| LH2b/LH2a | 2.5 | 2.25 | 25 | 3.375 | 2.11111 | 60 | 0.37466 |

**Supplementary Figure 14 │ Quantification of LH2a and LH2b mRNA levels in highly and poorly metastatic lung adenocarcinoma cell lines in Fig. 4e.** PCR bands were quantified using ImageJ and normalized on the basis of LH2b levels in 393P cells. p values indicate comparisons between highly and poorly metastatic groups (2-tailed Student's t test, n=2 or 4).

## Supplementary Figure 15 │ Uncut DNA agarose gel image for Figure 4g

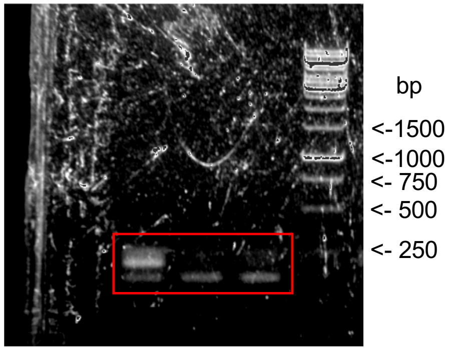

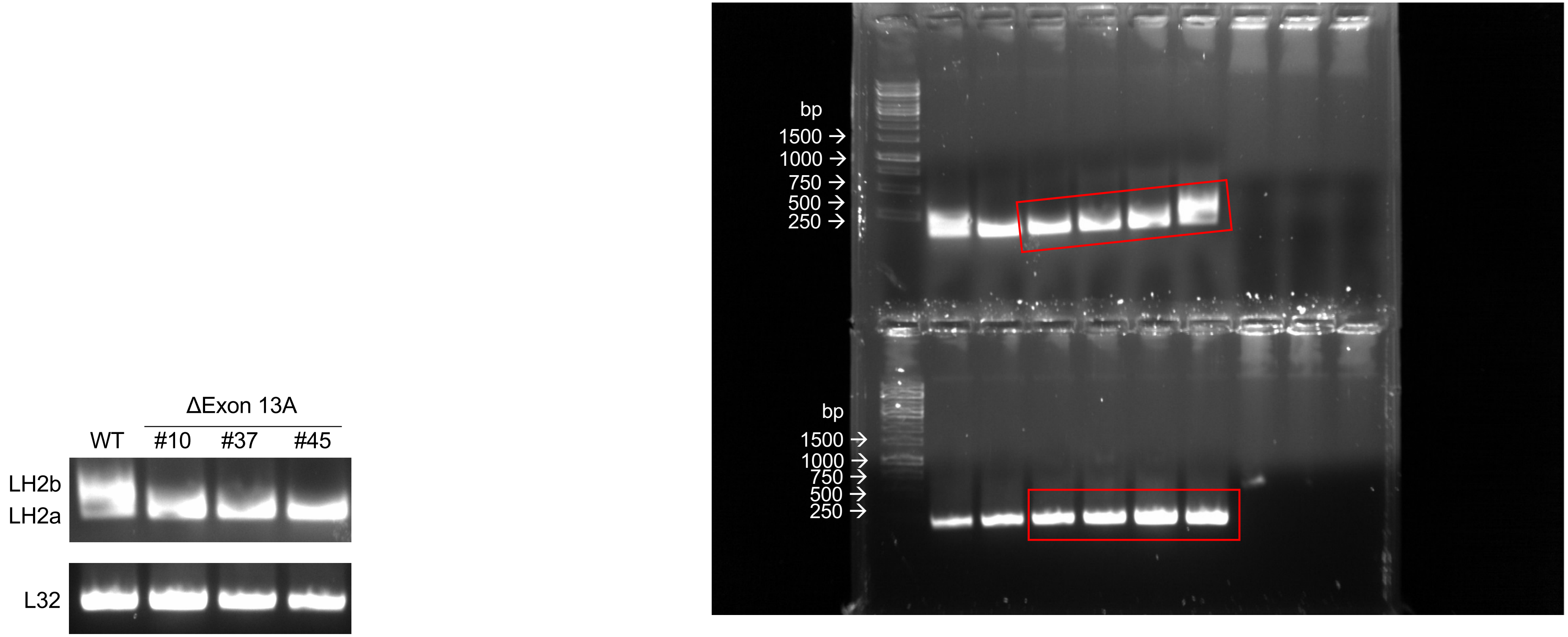


**Supplementary Figure 16 │ LH2b loss in 344SQ_Δexon13A cells. Reverse Transcriptase**-PCR analysis of LH2a and LH2b mRNA levels in parental (WT) and exon 13A-deleted 344SQ cells. Results are representative of replicate biological samples.

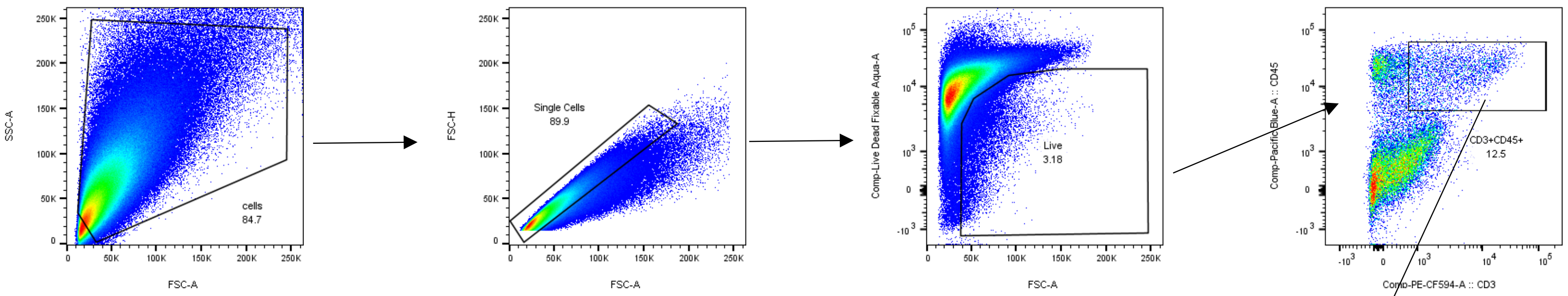


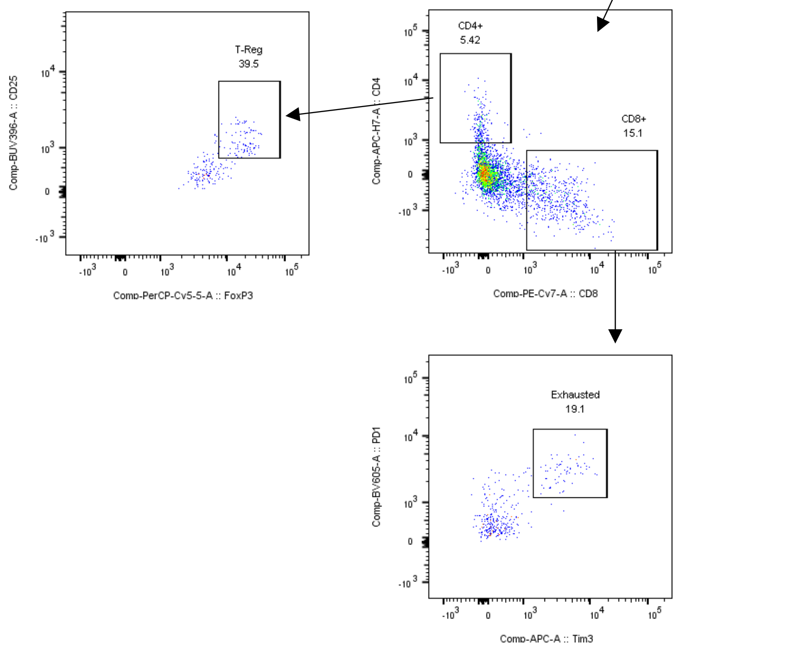


**Supplementary Figure 17 | Gating scheme utilized for the flow cytometry analysis of the T cell subsets from a representative tumor sample.** The lymphocytes were first gated using a dot plot with FSC versus SSC, and then exclusion of doublets using FSC-H versus FSC-A. The cells were stained for markers described in the materials and methods. Live cells were selected from negative BV510 populations. The T-cells were identified from double positive CD3+ and CD45+ gate. Specific CD4+ or CD8+ T-cells were gated from APC-Cy7 CD4 and PE-Cy7 CD8. Exhausted CD8 T-cells were gated from double positive PD1 and Tim3. These were gated from the original CD8+ T-cell gate. T-regulatory cells were gated using double positive CD25 and Tim3. These were gated from the original CD4+ T-cell gate.

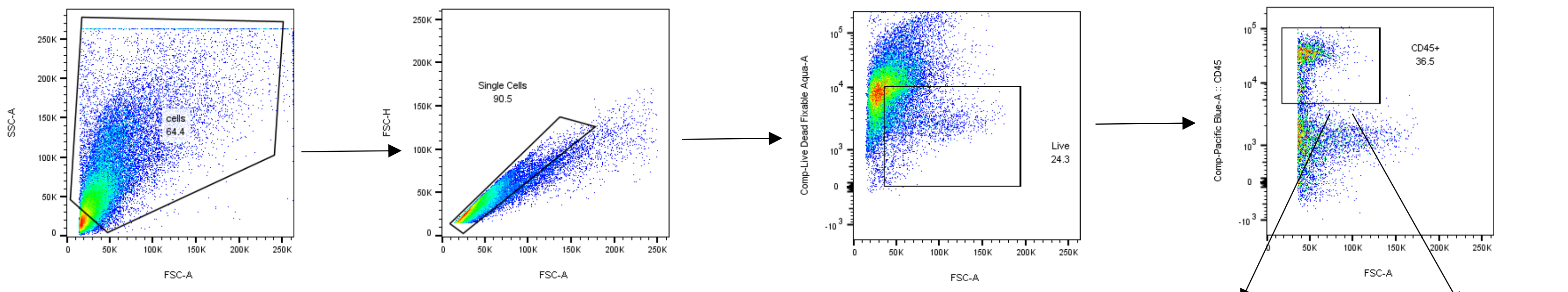


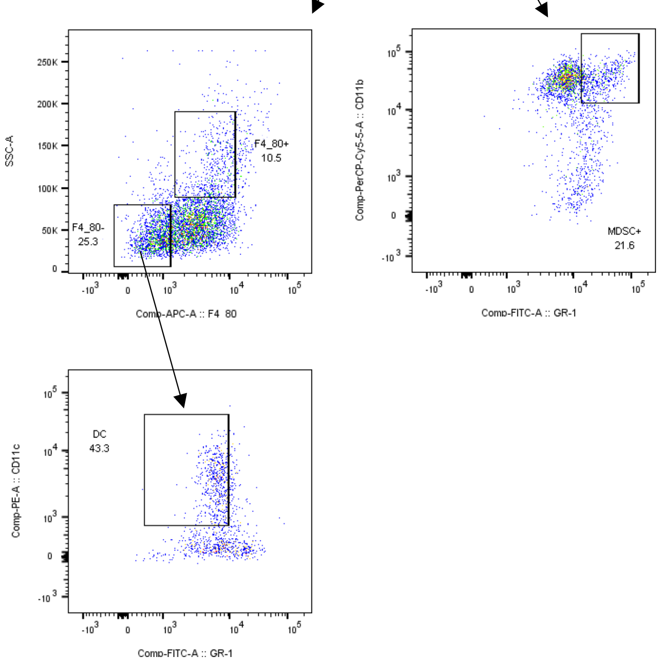


**Supplementary Figure 18 │ Gating scheme utilized for the flow cytometry analysis of myeloid cells from a representative tumor sample.** The cells were first gated using a dot plot with FSC versus SSC, and then exclusion of doublets using FSC-H versus FSC-A. The cells were stained for markers described in the materials and methods. Live cells were selected from negative BV510 populations. All CD45 positive cells were utilized to probe for myeloid subsets. From the CD45+ gate MDSC were gated from double positive Gr-1+ and CD11b++. The dendritic cell population was gated from F4/80 negative gate to a GR1 negative and CD11c positive gate.

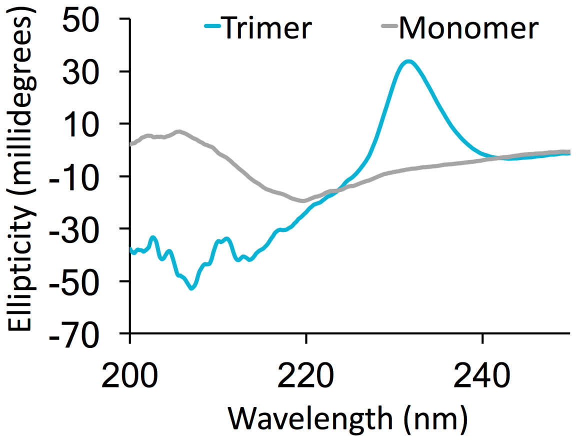


**Supplementary Figure 19 │ Trimeric synthetic telopeptide structure validation.** Trimeric and monomeric synthetic telopeptides were subjected to circular dichroism spectroscopy. Trimeric synthetic telopeptide (cyan) has a characteristic triple helix peak between 220 and 240 nm. Monomeric synthetic telopeptide (grey) lacks this peak. Results are mean values from replicate samples (n=2).

**Supplementary Table 1** DNA primers used in this study

| Name | Primer sequence |
|---|---|
| mPGGHG forward | GGTTCGGCTAGCATGGACGGCTCTGAAGATGACCCG |
| mPGGHG reverse | GCGTCGGCGGCCGCCTATGGGGATGACCTTTGTATCCGGCC |
| hLH1 forward | GGTGCGGATCCCCGGAGGACAACCTTTTAGTCCTC |
| hLH1 reverse | GCGTCGGCGGCCGCTTAGGGATCGACGAAGGAGACTGCGATG |
| hLH3 forward | GGTGCGGATCCGACCCGGTCAACCCAGAGAAGCTG |
| hLH3 reverse | GCGTCGGCGGCCGCTCAGGGGTCGACAAAGGACACCATG |
| hLH2 forward | GCGTCGTCTAGAAGCATCCCCACAGATAAATTATTAGTCATAACTGTAG |
| hLH2 reverse | GCGTCGGCGGCCGCTCAGGGATCTATAAATGACACTGCAATGTATCTTGTTCC |
| mPGGHG D300E forward | GGGGCCATATCTTCTGGGAGCAGGACATCTGGATGTTCC |
| mPGGHG D300E reverse | GGAACATCCAGATGTCCTGCTCCCAGAAGATATGGCCCC |
| LH1/LH2 swap forward | GTCGAGCAGCCTTGTCCAGATGTCTTCTGGTTTCCCATATTTACGGAGGCGGCCTGTGATGAGCTG |
| LH1/LH2 swap reverse | AAATATGGGAAACCAGAAGACATCTGG |
| hLH2 D115E forward | GGTTGTCATGTTTACTGAATGCTTTGAAGTCATATTTGCTGGTGGTCC |
| hLH2 D115E reverse | GGACCACCAGCAAATATGACTTCAAAGCATTCAGTAAACATGACAACC |
| hLH3 D112A forward | GGAGGATATGATCATCATGTTTGTGGCTAGCTACGACGTGATTCTGGCC |
| hLH3 D112A reverse | GGCCAGAATCACGTCGTAGCTAGCCACAAACATGATGATCATATCCTCC |
| hLH3 D115A forward | GATCATCATGTTTGTGGATAGCTACGCCGTGATTCTGGCCGGCAGC |
| hLH3 D115A reverse | GCTGCCGGCCAGAATCACGGCGTAGCTATCCACAAACATGATGATC |
| Mouse Exon 13a forward | CGATCTGAGATGAATGAAAGG |
| Mouse Exon 13a reverse | GCAGTTGATATCAGCCGTCC |
| mL32 forward | AGAGGACCAAGAAGTTCATCAG |
| mL32 reverse | CCAGCTCCTTGACATTGTGG |
| Human Exon 13a forward | CGATCAGAGATGAATGAAAGG |
| Human Exon 13a reverse | GCAGTGGATAATAGCCTTCC |
| hL32 forward | AGAGAACCAAGAAGTTCATCC |
| hL32 reverse | CAGCTCCTTGACGTTGTGG |
| hLH2-EP forward | CTGAATTTTGTAGTAAAATACTCCCCTGAAGAACAGCCTTCTCTTCGTCCTCATCATGATGC |
| hLH2-EP reverse | GCATCATGATGAGGACGAAGAGAAGGCTGTTCTTCAGGGGAGTATTTTACTACAAAATTCAG |
| hLH2 L735D forward | GGAGCTTCATGCATCCTGGGAGAGACACACATTTGCATGAAGGACTTCCTG |
| hLH2 L735D reverse | CAGGAAGTCCTTCATGCAAATGTGTGTCTCTCCCAGGATGCATGAAGCTCC |

**Supplementary Table 2** Guide RNAs and donor ssODN for CRISPR-Cas9 PLOD2 editing

| Name | Primer sequence |
|---|---|
| mLH2 Exon 13A gRNA1 | CAAGATGCCTCCCTCACACA |
| mLH2 Exon 13A gRNA2 | CCCCATAATAGCCTCCTTTG |
| hLH2 Exon 13A gRNA1 | CAAAGAATACCTGAGAGAGC |
| hLH2 Exon 13A gRNA2 | AGTAGTAAAGTTTCTTTAAG |
| mLH2 D689A gRNA | CGATGCCTCAACCTTTACCA |
| mLH2 D689A ssODN | CTCCTACATTATTCAGAGCAATGTTGATGGTAAAGGTCGACGCAGCGTGATGGGGACGGAGCGA-<br>-GCGCTGTCTTTCAGGTGAGTAC |
| mLH2 L735D gRNA | GGAGCTTCATGCATCCTGGG |
| mLH2 L735D ssODN | CACTGCAATGTATCGTGTTCCATTTTTAACAGGAAGTCCTTCGTGCAAATGTGTGTCTCTCCCGG-<br>-GATGCATGAAGCTCCAGCCTTTTCGTGGGGACTCAATGGAGCAATTATACCTC |
| mLH2 KO gRNA1 | GCCGGGATAGCCCGGGACGC |
| mLH2 KO gRNA2 | CTCTGCTTGCTGGTTTCCAC |
