# Supplementary material for: A collagen glucosyltransferase drives lung adenocarcinoma progression in mice
Source: Commun Biol. 2021 Apr 19;4:482. doi: 10.1038/s42003-021-01982-w (PMC8055892; doi:10.1038/s42003-021-01982-w)
Supplement: Supplementary file 3 — Description of Additional Supplementary Files [file 42003_2021_1982_MOESM3_ESM.pdf]

Description of Additional Supplementary Files

File name: Supplementary Data 1

Description: Underlying Data for main figures.
