# Supplementary material for: A collagen glucosyltransferase drives lung adenocarcinoma progression in mice
Source: Commun Biol. 2021 Apr 19;4:482. doi: 10.1038/s42003-021-01982-w (PMC8055892; doi:10.1038/s42003-021-01982-w)
Supplement: Supplementary file 5 — Reporting Summary [file 42003_2021_1982_MOESM5_ESM.pdf]

nature research

Corresponding author(s): Jonathan Kurie

Last updated by author(s): Feb 15, 2021

Reporting Summary

Nature Research wishes to improve the reproducibility of the work that we publish. This form provides structure for consistency and transparency in reporting. For further information on Nature Research policies, see our Editorial Policies and the Editorial Policy Checklist.

Statistics

For all statistical analyses, confirm that the following items are present in the figure legend, table legend, main text, or Methods section.

n/a	Confirmed	
☐	☒	The exact sample size (*n*) for each experimental group/condition, given as a discrete number and unit of measurement
☐	☒	A statement on whether measurements were taken from distinct samples or whether the same sample was measured repeatedly
☐	☒	The statistical test(s) used AND whether they are one- or two-sided<br>*Only common tests should be described solely by name; describe more complex techniques in the Methods section.*
☐	☒	A description of all covariates tested
☐	☒	A description of any assumptions or corrections, such as tests of normality and adjustment for multiple comparisons
☐	☒	A full description of the statistical parameters including central tendency (e.g. means) or other basic estimates (e.g. regression coefficient) AND variation (e.g. standard deviation) or associated estimates of uncertainty (e.g. confidence intervals)
☐	☒	For null hypothesis testing, the test statistic (e.g. *F*, *t*, *r*) with confidence intervals, effect sizes, degrees of freedom and *P* value noted<br>*Give P values as exact values whenever suitable.*
☒	☐	For Bayesian analysis, information on the choice of priors and Markov chain Monte Carlo settings
☒	☐	For hierarchical and complex designs, identification of the appropriate level for tests and full reporting of outcomes
☐	☒	Estimates of effect sizes (e.g. Cohen's *d*, Pearson's *r*), indicating how they were calculated

Our web collection on statistics for biologists contains articles on many of the points above.

Software and code

Policy information about availability of computer code

Data collection	Flow cytometry data were collected on BD LSR Fortessa (BD Biosciences); Cell aggregates were imaged with a Nikon A1 confocal microscope; circular dichroism spectra were measured using a J-810 spectropolarimeter.
Data analysis	Flow cytometry data were analyzed using FlowJo softweare (v.10.5.3; Tree Star); Cell projection length and RT-PCR results were manually quantified using ImageJ; Circular dichroism spectra were analyzed using Excel.

For manuscripts utilizing custom algorithms or software that are central to the research but not yet described in published literature, software must be made available to editors and reviewers. We strongly encourage code deposition in a community repository (e.g. GitHub). See the Nature Research guidelines for submitting code & software for further information.

Data

Policy information about availability of data

All manuscripts must include a data availability statement. This statement should provide the following information, where applicable:

- Accession codes, unique identifiers, or web links for publicly available datasets
- A list of figures that have associated raw data
- A description of any restrictions on data availability

Data sharing not applicable to this article as no datasets were generated or analyzed during the current study.

Field-specific reporting

Please select the one below that is the best fit for your research. If you are not sure, read the appropriate sections before making your selection.

- [x] Life sciences
- [ ] Behavioural & social sciences
- [ ] Ecological, evolutionary & environmental sciences

For a reference copy of the document with all sections, see nature.com/documents/nr-reporting-summary-flat.pdf

Life sciences study design

All studies must disclose on these points even when the disclosure is negative.

Sample size	The effect sample size was estimated based on prior studies using the same models.
Data exclusions	For orthotopic mouse tumor models, mice were excluded from the analysis if they died at the time of tumor cell injection due to hemorrhage or pneumothorax. For human lung cancer collagen glucosylation analysis, samples were excluded if they did not include both tumor and paired normal tissues. Samples used for collagen cross-link analysis were excluded if they had low collagen yield.
Replication	Measurements were taken from replicate biological samples. Mean values determined from replicate (n≥3) biological samples. Results are representative of replicate experiments.
Randomization	For mouse tumor models, mice were randomized to balance the cohorts on the basis of age.
Blinding	Whenever possible, investigators were blinded to the cohorts at the time of assessment.

Reporting for specific materials, systems and methods

We require information from authors about some types of materials, experimental systems and methods used in many studies. Here, indicate whether each material, system or method listed is relevant to your study. If you are not sure if a list item applies to your research, read the appropriate section before selecting a response.

Materials & experimental systems

n/a	Involved in the study	
[ ]	[x]	Antibodies
[ ]	[x]	Eukaryotic cell lines
[x]	[ ]	Palaeontology and archaeology
[ ]	[x]	Animals and other organisms
[x]	[ ]	Human research participants
[x]	[ ]	Clinical data
[x]	[ ]	Dual use research of concern

Methods

n/a	Involved in the study	
[x]	[ ]	ChIP-seq
[ ]	[x]	Flow cytometry
[x]	[ ]	MRI-based neuroimaging

Antibodies

Antibodies used	The antibodies (clone, dilution, company, catalogue #) used for flow cytometry are as follows: CD8 PE-Cy7 (53-6.7) l/800FCBiolegend/100721, CD3 PE-594 {17A2) 1/100 FCBiolegend/100246, CD4 APC-Cy7 (RM4-5) 1/100 FCBiolegend/100526, FoxP3 PerCp-Cy5.5 (FJK-16s) 1/100 FCeBioscience/45-5773-82, CD45 Pacific Blue (30-Fll) 1/100 FCBiolegend/103126, CD25 BUV395 (PC61) 1/100 FCBD Biosciences/564022, CD11c BV786 (N418) 1/100 FCBiolegend/117335, GR1 BV711 (RB6-8C5) 1/100 FCBiolegend/108443, TIM3 APC (B8.2c12) 1/100 FCBiolegend/134007, PD1 BV605 {29F.1A12) 1/100 FCBiolegend/135220, F4/80 APC (BM8.1) 1/100 FCTonbo/20-4801-U1O0, CD11b BV650 (M1170) 1/100 FCBiol egend/101239 , Ghost aqua BV510 1/50 FCTonbo.
Validation	Positive and negative controls and, when applicable, leave-one-out strategies were included in each flow cytometric analysis"

Eukaryotic cell lines

Policy information about cell lines

Cell line source(s)	344SQ cells derived from K-ras/Tp53-mutant mice were generated in-house; MC3T3-El and H358 cells were obtained from ATCC.
Authentication	344SQ cells are authenticated on the basis of sequencing to confirm Kras and p53 mutational status.

Mycoplasma contamination	All cell lines were tested negative for mycoplasma.
Commonly misidentified lines (See ICLAC register)	N/A

Animals and other organisms

Policy information about studies involving animals; ARRIVE guidelines recommended for reporting animal research

Laboratory animals	Immunocompetent 129/ Sv mice syngeneic to 344SQ cells were bred in-house and nude mice were ordered from The University of Texas MD Anderson Cancer Center ERO department.
Wild animals	N/A
Field-collected samples	N/A
Ethics oversight	All mouse studies were approved by the Institutional Animal Care and Use Committee at The University of Texas MD Anderson Cancer Center. The use of lung tissues quantification of collagen cross-links in this study was performed under Institutional Review Board - approved protocol IRB(2)0910-01565x at Houston Methodist Research Institute.

Note that full information on the approval of the study protocol must also be provided in the manuscript.

Flow Cytometry

Plots

Confirm that:

- [x] The axis labels state the marker and fluorochrome used (e.g. CD4-FITC).
- [x] The axis scales are clearly visible. Include numbers along axes only for bottom left plot of group (a 'group' is an analysis of identical markers).
- [x] All plots are contour plots with outliers or pseudocolor plots.
- [x] A numerical value for number of cells or percentage (with statistics) is provided.

Methodology

Sample preparation	Cell samples were collected from primary murine subcutaneous tumor tissues, digested with collagenase/dispase solution, and passed through single-cell filters.
Instrument	FACS data werecollected using BD LSRFortessa flow cytometer.
Software	FACS data were collected using FACSDiva and analyzed using FlowJo.
Cell population abundance	FACS analysis was performed on each sample to a total cell number between 500,000 to 2,000,000 events with a threshold of 10,000 to increase quality of samples per event. Each gated population was sorted so that at least 1,000 cells for the furthest gated cell population was recorded to obtain satisfactory percentage of the cell population. FACS quality was also ensured using compensation controls and FMO controls to verify that observed and gated populations were accurate and distinct."
Gating strategy	All samples were gated for FSC/SSC, then gated for FSC-A/FSC-H, then gated for CD45+ cells. For T-cell populations, CD45+ were double-gated for CD3+ cells. CD45+CD3+ cells were then gated for CD4+ or CD8+ cells from the same populations. CD8+ cells were gated for the indicated populations listed in the graphs from the figures. For antigen presenting populations, CD45+ cells were gated for the populations indicated on the graphs in the figures. Gates were drawn from distinct, observable stained populations using prior FMO controls to indicate where to draw the positive gates that were separate from the negative populations.

- [x] Tick this box to confirm that a figure exemplifying the gating strategy is provided in the Supplementary Information.
